# Spatial Mapping of Stereoisomeric and Isobaric Alkaloids in *Mitragyna speciosa* Tissues by High-Resolution DESI-cIM-MS

**DOI:** 10.1021/acs.analchem.5c04730

**Published:** 2025-11-14

**Authors:** Pattipong Wisanpitayakorn, Adchata Konsue, Thanutchaporn Sartyoungkul, Ammarin In-on, Yongyut Sirivatanauksorn, David R. Gang, Prasat Kittakoop, Sakda Khoomrung

**Affiliations:** † Siriraj Center of Research Excellence in Metabolomics and Systems Biology (SiCORE-MSB), Faculty of Medicine Siriraj Hospital, 65106 Mahidol University, Bangkok 10700, Thailand; ‡ Siriraj Metabolomics and Phenomics Center, Faculty of Medicine Siriraj Hospital, Mahidol University, Bangkok 10700, Thailand; § Thailand Metabolomics Association, Bangkok 10700, Thailand; ∥ Institute of Biological Chemistry, 6760 Washington State, University, Pullman, Washington 99164, United States; ⊥ Chulabhorn Graduate Institute, Program in Chemical Sciences, Laksi, Bangkok 10210, Thailand; # 67969 Chulabhorn Research Institute, Laksi, Bangkok 10210, Thailand; ¶ Department of Biochemistry, Faculty of Medicine Siriraj Hospital, Mahidol University, Bangkok 10700, Thailand; ∇ Center of Excellence for Innovation in Chemistry (PERCH−CIC), Faculty of Science Mahidol University, Bangkok 10400, Thailand

## Abstract

Conventional mass spectrometry imaging (MSI), even when combined with low-resolution ion mobility, lacks the resolving power to distinguish stereoisomers. To address this limitation, we developed a high-resolution desorption electrospray ionization cyclic ion mobility mass spectrometry (DESI-cIM-MS) method for in situ separation and spatial mapping of stereoisomeric compounds, using *Mitragyna speciosa* (kratom) as a model system. We characterized and validated the separation of four mitragynine-type stereoisomers—mitragynine (MG), speciogynine (SG), mitraciliatine (MC), and speciociliatine (SC)—using chemical standards. Notably, SC exhibited two gas-phase conformers, fast (SC-F) and slow (SC-S), supported by quantum chemical calculations. Using multipass separation and targeted ion slicing, we resolved and mapped SG, MC, and SC-S in surface-spotted standards. To address coelution between MG and SC-F, we developed a pixel-wise subtraction strategy based on the SC-F/SC-S intensity ratio to mitigate SC-F interference in the MG ion image. Direct analysis of kratom twig tissue revealed distinct spatial distributions for each stereoisomer. MG was found broadly throughout the twig except in the xylem. MC was concentrated in the pith, with some presence in the bark. SC and SG were predominantly localized in the bark, especially the epidermis. Furthermore, we resolved two additional important alkaloids, paynantheine and 7-OH-mitragynine, from their isobaric compounds; both were distributed throughout the twig except the xylem. These findings demonstrate the importance of high-resolution ion mobility in MSI for accurately resolving structurally similar compounds and improving spatial analysis in metabolomics and natural product research.

## Graphical abstract

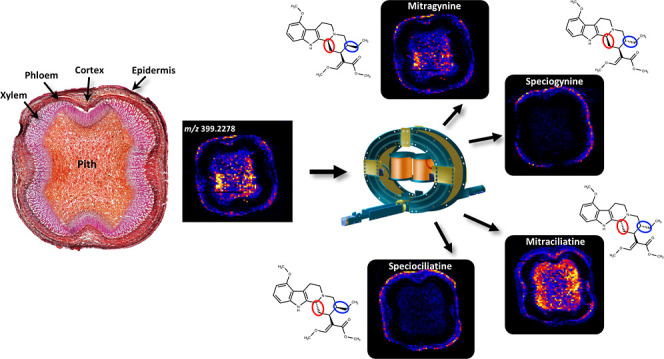

## Introduction

Stereoisomers pose a persistent challenge in mass spectrometry (MS)-based analysis due to their identical molecular weights and often indistinguishable fragmentation patterns.^1^ This challenge is especially pronounced in mass spectrometry imaging (MSI), where compounds must be resolved and localized directly from complex biological tissues without chromatographic separation.^2^ Ion mobility spectrometry (IMS) offers a powerful means of enhancing MS selectivity by separating ions based on their size, shape, and charge in the gas phase.^3,4^ However, low-resolution IMS often lacks the resolving power required to distinguish closely related stereoisomers,^5,6^ particularly in high-complexity samples like plant tissue. Recent advances in cyclic ion mobility mass spectrometry (cIM-MS), which allow ions to traverse extended path lengths over multiple passes, have substantially increased ion mobility resolution and opened new opportunities for structural isomer separation in untargeted analyses.^2,7,8^

One class of compounds that exemplifies this challenge is the indole alkaloids of *Mitragyna speciosa* (kratom), a medicinal plant native to Southeast Asia.^9,10^ Kratom biosynthesizes a suite of stereoisomeric alkaloids, including mitragynine (MG), speciogynine (SG), speciociliatine (SC), and mitraciliatine (MC), that differ only in the spatial configuration at three stereocenters but exhibit distinct pharmacological profiles.^11^ While prior studies have employed chromatographic methods to resolve these isomers in extracts,^12–14^ no mass spectrometry imaging approach has yet enabled separation and spatial localization of such complex stereoisomers directly from intact tissue.

In this study, we developed a desorption electrospray ionization cyclic ion mobility mass spectrometry (DESI-cIM-MS) method that enables in situ separation and spatial mapping of stereoisomeric and isobaric alkaloids in kratom tissue. By combining multipass cyclic ion mobility, targeted ion slicing, and pixel-wise subtraction, we achieve conformer- and isomer-resolved imaging of mitragynine-type and other pharmacologically relevant alkaloids in intact kratom twig tissue. Together, these results establish a generalizable platform for analyzing structurally similar small molecules in complex biological samples and expand the utility of MSI in spatial metabolomics and natural product study.

## Experimental Section

### Chemicals and Reagents

Chemical standards (purity >98%) of MG (CAS No. 4098-40-2, Cat. No. 11151), SC (CAS No. 14382-79-7, Cat. No. 27246), MC (CAS No. 14509-92-3, Cat. No. 39856), paynantheine (CAS No. 4697-66-9, Cat. No. 21841), and 7-OH-mitragynine (CAS No. 174418-82-7, Cat. No. 13114) were purchased from Cayman Chemical (Ann Arbor, MI, USA). SG (purity >98%) (CAS No. 4697-67-0, Cat. No. CDX-00019296) was obtained from ChromaDex (Irvine, CA, USA). LC/MS-grade methanol was sourced from DKSH Technology Limited (Bangkok, Thailand), and ultrapure water was produced using a Milli-Q system (Millipore, Billerica, MA, USA).

### Sample Collection and Preparation

Twig tissue from a mature *M. speciosa* tree was collected in Chumphon Province, Thailand, in May 2024 for method validation. A ∼2 cm-thick transverse segment was excised from a mature secondary branch, rinsed sequentially with tap water and autoclaved ultrapure water to remove surface debris, then wrapped in aluminum foil and transported at ∼4 °C in a sealed container with cooling gel packs. Upon arrival at the laboratory, a 1 cm × 1 cm section of the twig was trimmed and embedded in 8% (w/v) porcine gelatin (G1890, Sigma-Aldrich). The embedded sample was snap-frozen in liquid nitrogen and stored at −80 °C until cryosectioning. For DESI-cIM-MS analysis, the sample was cryosectioned at 30 μm thickness using a cryostat maintained at −15 °C. Sections were thaw-mounted onto precleaned, uncoated glass microscope slides and subsequently dried in a desiccator for 1 h prior to analysis.

### Quantum Chemical Calculations

To investigate the gas-phase conformational landscape of the mitragynine-type diastereomers, we performed quantum chemical calculations using the Gaussian 03 software package.^15^ Initial structures for MG, SG, SC, and MC were generated in their protonated forms, with the proton placed on the tertiary nitrogen—consistent with protonation states expected under positive-mode electrospray ionization (ESI^+^) conditions. Geometry optimizations were carried out using the B3LYP functional in combination with the 6-31G* basis set.^16,17^ For each stereoisomer, eight initial conformers were subjected to full geometry optimization. All resulting structures were confirmed as local minima by verifying the absence of imaginary vibrational frequencies.

### Direct Infusion cIM-MS for Arrival Time Characterization

To investigate the multipass arrival time behavior of mitragynine stereoisomers, direct infusion experiments were performed on a SELECT SERIES Cyclic IMS instrument (Waters, USA) using positive electrospray ionization (ESI^+^), following a protocol previously established in our earlier study.^2^ Each chemical standard was prepared at a concentration of 4 μM and infused at a flow rate of 10 μL/min. Data were acquired in high-definition MS (HDMS) mode over an *m*/*z* range of 50–1200 Da at a scan rate of 1 scan/s. The following cIM-MS parameters were applied: 2 pushes per bin, 22 V static traveling wave (TW) height, 375 m/s cyclic TW velocity, 375 m/s array TW velocity, TW ramp start and end heights of 15 and 35 V, respectively, ramping rate of 2.5 V/ms, injection time of 10 ms, and a combined ejection and acquisition time of 26.4 ms. Multipass arrival times were obtained by manually adjusting the separation time. Peak picking and data processing were performed using High-Definition Imaging (HDI) software (v1.8, Waters, USA) and DriftScope software (v3.0, Waters, USA), and in-house developed Python scripts.

### Standard Spotting for Method Validation

To validate stereoisomer separation, spotting experiments were performed using MG, SG, SC, and MC. Each compound was prepared at a concentration of 100 μM in 2:1 (v/v) water/methanol. For each compound, 2.5 μL of the solution was manually spotted side by side onto a precleaned glass microscope slide. The slide was dried in a desiccator for approximately 1 h before analysis. Dried spots were analyzed by DESI-cIM-MS using the same acquisition parameters as applied to tissue sections.

### DESI-cIM-MS Analysis of Plant Tissues

Sectioned plant tissues were analyzed in ESI^+^ using DESI-cIM-MS. The DESI spray solvent consisted of methanol–water (98:2, v/v) with 0.1% formic acid. Acquisition parameters were as follows: capillary voltage, 0.7 kV; sampling cone voltage, 40 V; source temperature, 150 °C; transfer line temperature, 350 °C; DESI gas pressure, 0.07 MPa; solvent flow rate, 1.0 μL/min; step size, 50 μm; and scan time, 1.0 s. Cyclic ion mobility separation was performed under conditions optimized from the direct infusion experiments.

Raw data were processed using HDI software with the following settings: HDMS experiment type, top 3000 most intense peaks, low energy threshold of 10 counts, *m*/*z* range of 50–1200, *m*/*z* window of 0.02 Da, and MS resolution of 20,000. Drift parameters included drift/quad start at 1 bin and stop at 200 bins, drift/quad window of 1 bin, and minimum peak width of 2 bins. Lock-mass correction was applied using *m*/*z* 399.2278 (protonated MG) with a tolerance of ±0.25 Da, sampled every 5 min for 10 s. Total ion mobiligrams and mass spectra were further visualized and processed using DriftScope software.

## Results and Discussion

### Overview of the DESI-cIM-MS Workflow for Resolving Stereoisomers and Isobars

Conventional MSI struggles to resolve structurally similar small molecules such as the four diastereomeric alkaloids in *M. speciosa*—MG, SG, SC, and MC—which share identical *m*/*z* (399.2278 [M + H]^+^) and highly similar MS/MS spectra (Figure 1). To address this, we developed a stepwise DESI-cIM-MS workflow for the in situ separation and spatial mapping of stereoisomeric and isobaric small molecules. The workflow begins with DI-cIM-MS to characterize the ion mobility behavior of each stereoisomer and assess the potential for gas-phase conformer separation. Separation performance of the multipass and targeted ion slicing strategies was then assessed and optimized using surface-spotted standards under controlled conditions. For cases where physical separation was not achievable, we proposed a pixel-wise subtraction strategy to mitigate overlapping ion signals. The complete method was ultimately applied to intact kratom twig tissue to assess alkaloid localization in a native biological matrix.

**Figure 1:**
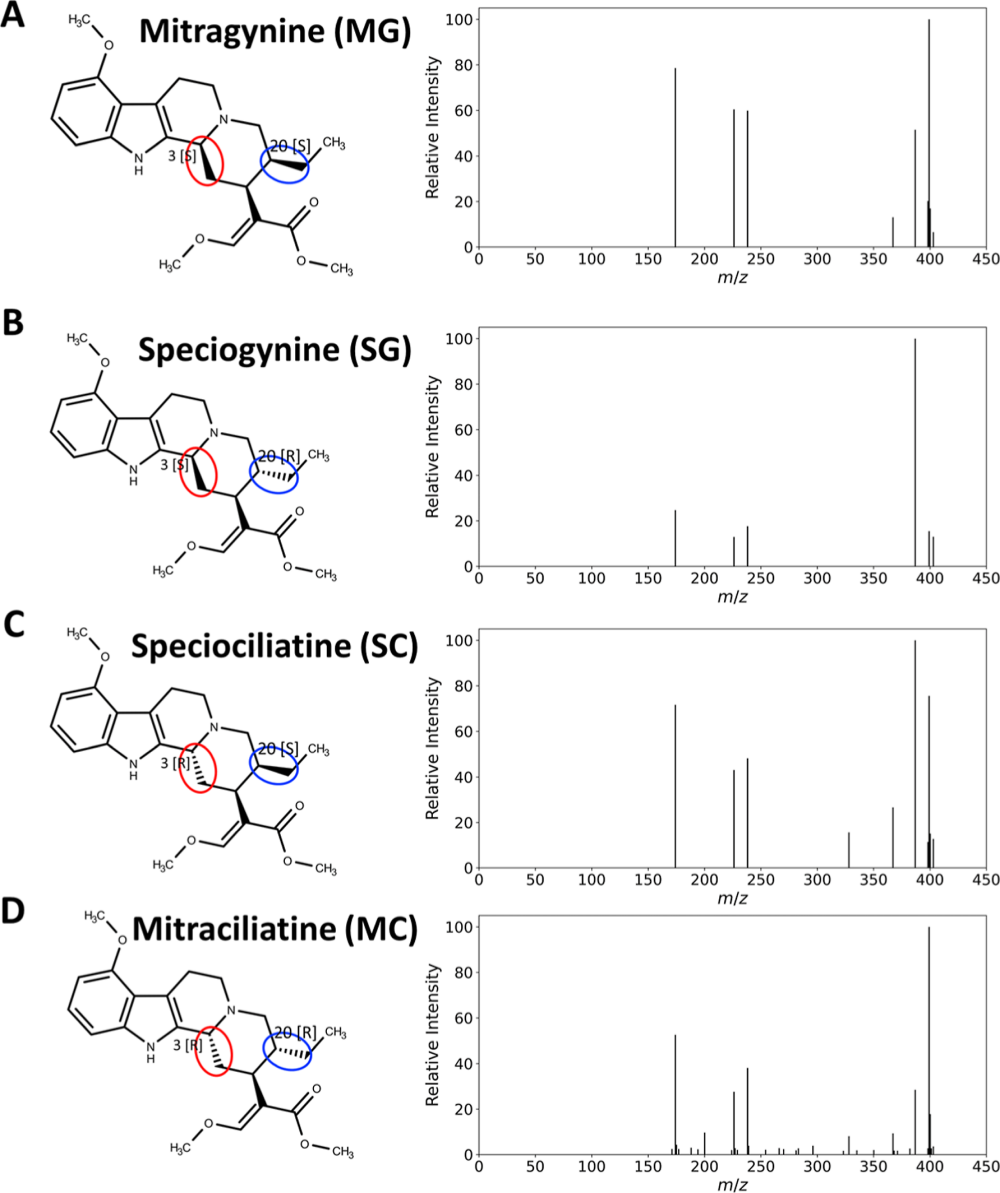
Structures and MS/MS fragmentation patterns of mitragynine and its stereoisomers acquired at 30 V collision energy. (A) Mitragynine (MG). (B) Speciogynine (SG, differs from MG at position 20 [*R*]^12^). (C) Speciociliatine (SC, differs from MG at position 3 [*R*]). (D) Mitracilliatine (MC, differs from MG at both positions 3 [*R*] and 20 [*R*]). Each panel shows the chemical structure (left) and corresponding MS/MS spectrum (right). All compounds share highly similar fragmentation patterns due to their identical core scaffolds and functional groups. They differ from mitragynine only in the configuration of one (speciogynine and speciociliatine) or two (mitraciliatine) stereocenters.

### Characterization of Mitragynine-Type Diastereomers by DI-cIM-MS

To characterize the arrival times of each stereoisomer, we first performed DI-cIM-MS on individual chemical standards to obtain their arrival time distributions (ATDs). The DI-cIM-MS data of the four stereoisomers were collected within 2 consecutive days to minimize the between-run variations. From these experiments, we obtained *n*-pass arrival times (*t*_*n*_) and the zero-pass drift time (*t*_0_), representing the time an ion takes to traverse the ion mobility section without completing any passes. The periodic arrival time (*t*_p_) was then calculated using the linear relationship *t*_*n*_ = *t*_0_ + *n* × *t*_p_.^18,19^ The *t*_p_ and *t*_0_ for the four stereoisomers are summarized in Table S1. Figure 2A present an overlay of their ATDs under 1-, 7-, and 16-pass conditions. We also measured the full width at half-maximum (FWHM) of each ATD peak as a function of the number of passes (Figure 2B). The increase in FWHM per pass, along with the widening gap between the centers of arrival time peaks, can be used to estimate the number of passes required for compound separation. Initial DI-cIM-MS analyses revealed that MG, SG, and MC each produced a single, well-defined ATD peak. Unlike the other stereoisomers, SC consistently exhibited two gas-phase conformers, fast (SC-F) and slow (SC-S), which became baseline-resolved after seven passes (equivalent to a 7 m drift path). Each conformer was then isolated by targeted ion slicing and fragmented in the cIM transfer region at 30 V, yielding identical fragment ions with only minor differences in relative abundance (Figure S1).

**Figure 2:**
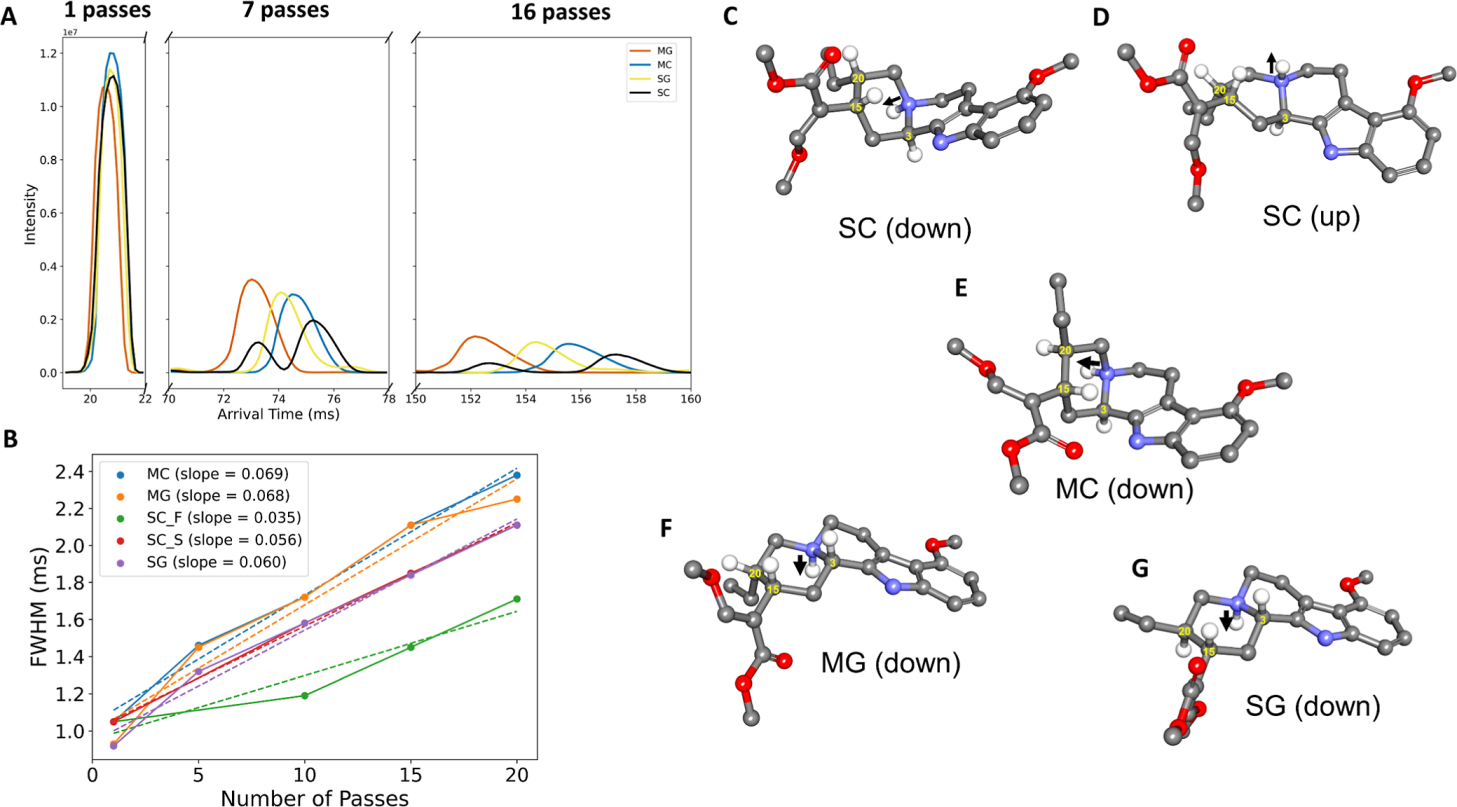
Gas-phase separation and conformational analysis of mitragynine-type diastereomers. (A) Arrival time distributions (ATDs) of MG, MC, SG, and SC at 1, 7, and 16 passes in cyclic ion mobility spectrometry (cIM-MS). SC exhibits a reproducible dual-peak profile corresponding to two gas-phase conformers: SC-F (fast) and SC-S (slow). (B) Full width at half-maximum (FWHM) values plotted against pass number for each compound, with linear fits represented by dotted lines. (C–G) Optimized gas-phase geometries of each protonated compound based on DFT calculations. SC exhibits two stable conformers: downward (C) and upward (D) N–H orientations. In contrast, MC (E), MG (F), and SG (G) adopt only a single stable conformation with the N–H vector directed downward, and no upward conformers were located as energy minima. Arrows indicate the direction of the protonated amine (adduct) at position 4 in each structure.

The reproducible dual-peak profile observed for SC indicates the presence of two kinetically stable gas-phase conformers separated by a substantial energy barrier, sufficient to prevent interconversion on the time scale of ion mobility separation. Quantum chemical calculations on SC support this interpretation, revealing two low-energy conformers that differ in the orientation of the N–H bond at the protonated tertiary nitrogen at position 4 in the structure: one with the proton directed above the molecular plane (downward; Figure 2C) and the other below it (upward; Figure 2D). Although the downward conformer is thermodynamically favored, the upward conformer lies only 2.80 kcal/mol higher in energy (Table S2), consistent with its persistence under ambient gas-phase conditions.^20,21^ In contrast, MC favors a single stable geometry, with the upward conformation ∼10.1 kcal/mol higher in energy (Figure 2E), making its population negligible under these experimental conditions. For MG and SG, only the downward N–H orientation was located as a true minimum (Figure 2F,G), and no stable upward conformer could be identified. Together, these results provide a structural explanation for the conformer-resolved ion mobility features observed for SC and highlight the ability of ion mobility spectrometry to detect subtle gas-phase conformational differences in moderately sized natural products.

### Development of a Cyclic IMS Separation Strategy for Mitragynine Diastereomer Mixtures

To develop an effective separation strategy for mitragynine-type stereoisomers, we performed DI-cIM-MS analysis on a mixture of their chemical standards. As shown in Figure 3A, under low-pass conditions, all four isomers produced a single, overlapping ATD peak. Initial attempts to improve resolution by simply increasing the number of passes were limited by two key challenges: (1) ∼2–5% progressive loss of signal intensity due to cumulative transmission losses for each increasing pass number,^7^ and (2) wrap-around effect in high pass number due to broadening of ATDs.^22^ After 16 passes (Figure 3A) (140 ms separation time), the ATDs of the mixture nearly filled the 98 cm cyclic IMS chamber, and further passes induced a wrap-around effect where early arriving ions, such as MG and SC-F, overlapped with later-arriving ions like MC and SC-S.

**Figure 3:**
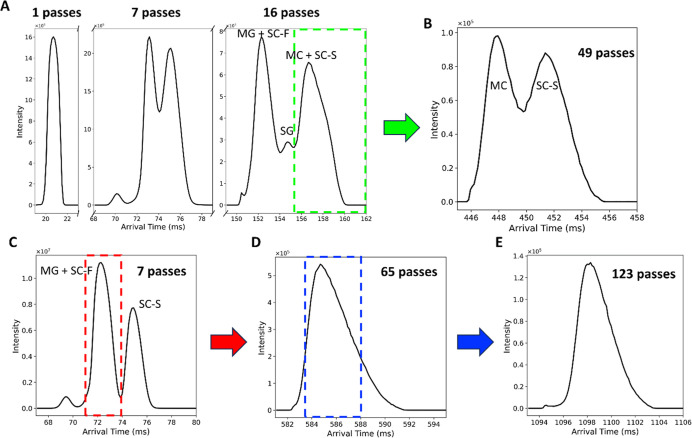
Cyclic ion mobility separation strategy for mitragynine-type stereoisomers. (A) Arrival time distributions (ATDs) of a four-isomer mixture, MG, SC, SG, and MC, collected at 1, 7, and 16 passes. SG was resolved after 16 passes. (B) To isolate MC and SC-S, only the ions within the green box in panel A were retained, while early arriving ions, corresponding to MG, SC-F, and SG, were discarded. The remaining ions were further separated to 49 passes, yielding improved separation. (C–E) Targeted separation of MG and SC-F demonstrated using a mixture containing only those two reference standards. (C) After 7 passes, only the central ATD segment containing MG and SC-F was retained (red box). (D) Continued separation to 65 passes showed no discernible peak splitting. (E) The central 4 ms of the coeluted peak was further isolated (blue box in D) and subjected to extended separation. After 123 passes, MG and SC-F remained unresolved.

Despite these physical constraints, SG was partially resolved from the remaining stereoisomers after 16 passes (Figure 3A). To further separate MC and SC-S, we employed a targeted ion slicing strategy by discarding the first 6 ms of ions inside the cIM chamber, thereby eliminating MG, SC-F, and SG. The remaining ions were subjected to an additional 33 passes (289.5 ms separation time), resulting in a clear resolution between MC and the SC-S after a total of 49 passes (Figure 3B).

However, separation of MG from SC-F could not be achieved using this approach, as their arrival times remained closely overlapping after 16 passes. To confirm the inability to resolve MG and SC-F, we performed DI-cIM-MS on a mixture of these two chemical standards. To mitigate the effects of signal broadening and wrap-around, we retained only the peak containing MG and SC-F after 7 passes and ejected the rest (Figure 3C). We then continued the separation up to 65 total passes, at which point the peak approached the spatial limits of the cIM device, yet no discernible separation between MG and SC-F was observed (Figure 3D). To further test the limit of resolution, we isolated only the central 4 ms of the coeluted peak—excluding both tailing regions—and subjected this narrow window to extended ion mobility separation. Although this approach sacrificed signal intensity, it minimized wrap-around and allowed continued separation. After 123 passes, MG and SC-F remained unresolved (Figure 3E). From prior analysis of individual chemical standards, we observed that the centroid arrival time gap between MG and SC-F increased by ∼0.032 ms per pass, while their combined FWHM broadened by ∼0.103 ms per pass (∼0.068 ms/pass for MG and ∼0.035 ms/pass for SC-F) (Figure 2B). Since the increase in FWHM exceeded the increase in arrival time gap, we concluded that MG and SC-F could not be resolved under our cIM-MS conditions.

### Assessing Stereoisomer Separation Using Surface-Spotted Standards

To validate our cIM-MS separation strategy under DESI imaging conditions, each stereoisomer was deposited side by side as small, desiccated droplets on a glass slide (Figure 4A), serving as a reference-standard spotting experiment to confirm consistent separation performance. We first applied a 16-pass ion mobility condition to assess the spatial resolution of SG. Following data processing with HDI software, the ion image of SG appeared well localized. However, a faint signal from the MC spot (the rightmost spot) was observed in the SG ion image (Figure 4B). To achieve clean SG signal, we ejected the coeluded peak of MC and SC-S. Then, the retained peaks of MG, SC-F, and SG were subjected to additional 30 passes. With this approach, we achieve a clean separation of SG from the minor MC signal (Figure 4C). To achieve clean ion images for MC and SC-S, the same cIM-MS settings previously optimized in the DI experiment were applied. This approach enabled successful differentiation and spatial localization of SC-S and MC (Figure 4D,E), while MG and SC-F remained unresolved (Figure 4F), consistent with our ESI-based results.

**Figure 4:**
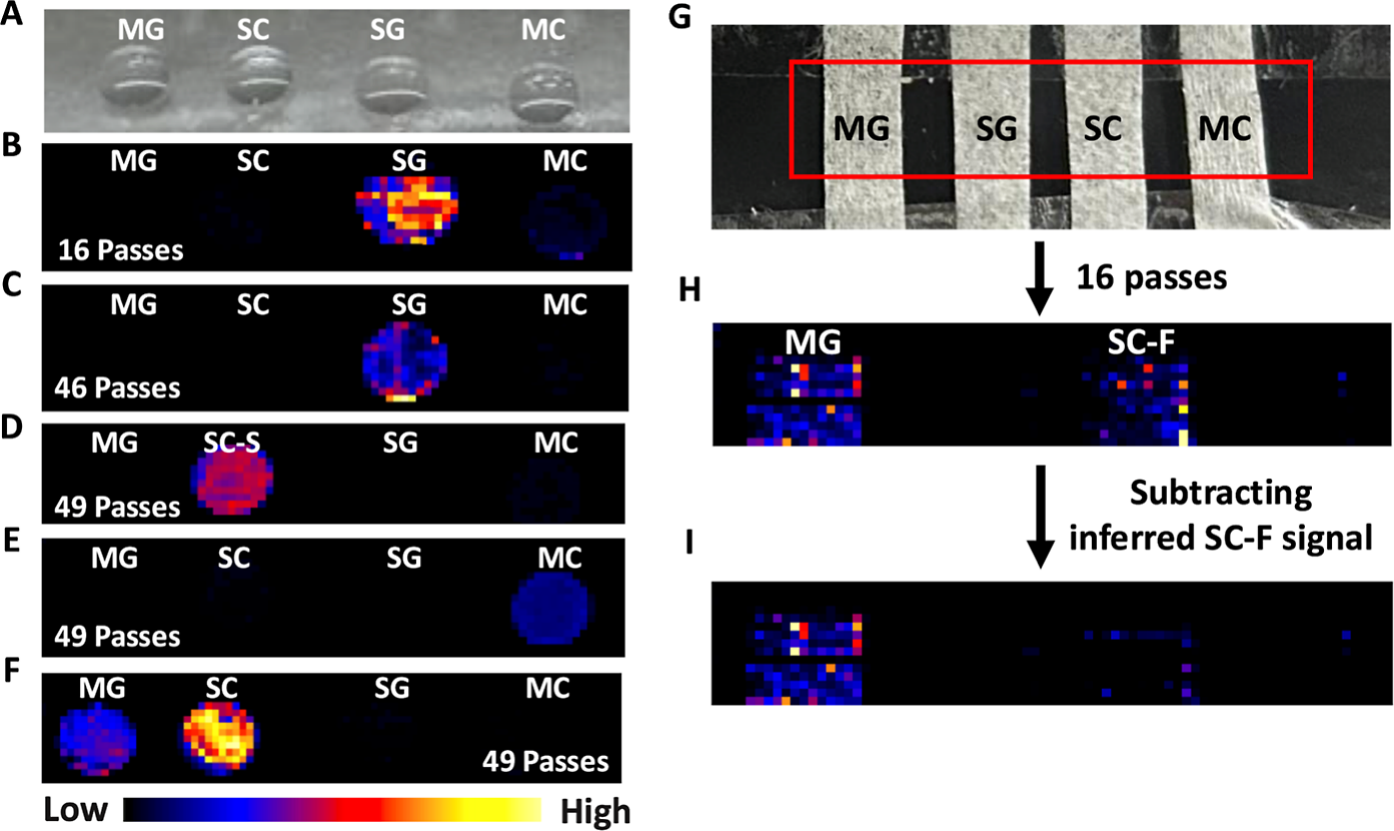
Validation of DESI-cIM-MS-based separation strategy of stereoisomers. (A) Four stereoisomers were spotted side by side on a glass slide to assess spatial separation. (B) A 16-pass cIM-MS method resolved SG, though a faint signal from the MC spot (the rightmost spot) was detected in the SG ion image. (C) Clean separation of SG was achieved by ejecting the overlapping MC/SC-S peak, followed by 30 additional passes on the retained peaks (MG, SC-F, SG). (D,E) The same cIM-MS settings used in DI-cIM-MS enabled clean ion images of MC and SC-S, though MG and SC-F remained unresolved. (G) The four stereoisomers were also spotted onto separate strips of cellulose-based lab wipes (Kimwipes) affixed to a glass slide, serving as a plant tissue mimic. (H) MG and SC-F coeluted under 16-pass conditions. (I) A cleaner MG ion image was obtained by pixel-wise subtraction of the estimated SC-F signal, based on its known distribution.

### Development of a Pixel-Wise Subtraction Strategy to Mitigate SC-F Interference

Because MG and SC-F cannot be resolved by cIM-MS under current conditions, we developed a pixel-wise subtraction strategy to approximate the spatial distribution of MG. As SC-F and SC-S are gas-phase conformers, they are expected to share the same spatial localization. Additionally, under our DESI conditions, the signal intensity ratio between SC-F and SC-S was observed to be approximately 1:1, rather than 0.52:1 as seen in the DI data (Figure 2A), likely due to differences in ionization and desorption mechanisms between DESI and ESI that influence the conformer populations formed during the ionization process. Our pixel-wise subtraction strategy involves two sequential acquisitions of the same tissue region: (1) a 16-pass cIM-MS run, and (2) a targeted ion slicing acquisition isolating SC-S and MC. We verified that alkaloids in kratom plant tissue can be measured twice with DESI-cIM-MS with only slight (∼10–20%) decrease in signal intensity, confirming the feasibility of this dual-acquisition approach.

To demonstrate the method in a controlled setting, we spotted the four stereoisomers onto strips of Kimwipes (Kimtech Science) affixed to a glass slide (Figure 4G). These cellulose strips served as a tissue mimic, as direct spotting onto glass resulted in compound washout after a single DESI scan. Under 16-pass conditions, MG and SC-F coeluted (Figure 4H). To normalize intensity differences between the two acquisitions, we calculated the ratio of combined SC-S and MC signals across data sets (0.76 in this case), accounting for signal differences due to resampling and variations in acquisition strategies. The SC-S ion map from the targeted ion-slicing acquisition was then scaled and multiplied by the experimentally determined SC-F/SC-S ratio (1:1 under our DESI conditions) to generate an estimated SC-F ion distribution. Because the ratio equals 1, this scaling step does not alter the intensity but is included to maintain methodological consistency with other data sets. This estimated SC-F map was subtracted pixel by pixel from the combined MG and SC-F signal in the 16-pass data set, yielding an approximated MG ion image. As shown in Figure 4I, this approach reduced SC-F interference by 81%, producing a cleaner MG signal. While the pixel-wise subtraction strategy does not fully eliminate SC-F signal, it provides a practical workaround when physical separation is not achievable. Complementary strategies, such as chemical derivatization and enzymatic tagging, may aid in further resolving coeluting species.

### Application of DESI-cIM-MS in Kratom Twig Tissue Section

To demonstrate our DESI-cIM-MS workflow in native biological contexts, we applied it to transverse twig cross sections from a mature *M. speciosa* tree. A 2 μL dried spot of kratom extract was first used to calibrate and fine-tune the ion mobility separation windows. All four mitragynine-type stereoisomers were successfully detected in situ, demonstrating that our optimized cIM-MS conditions are transferable from reference standards to native tissue environments. As shown in Figure 5, ion mobility-resolved MSI enabled clean separation and mapping of each isomer’s spatial localization, which would otherwise be conflated into a single *m*/*z* 399.2278 signal using conventional DESI-MS (Figure 5B). Although the MG map in Figure 5C includes pixel-wise subtraction of overlapping SC-F signal, this correction had minimal impact due to the low abundance of SC (∼5% of MG) in this particular section of tissue.

**Figure 5:**
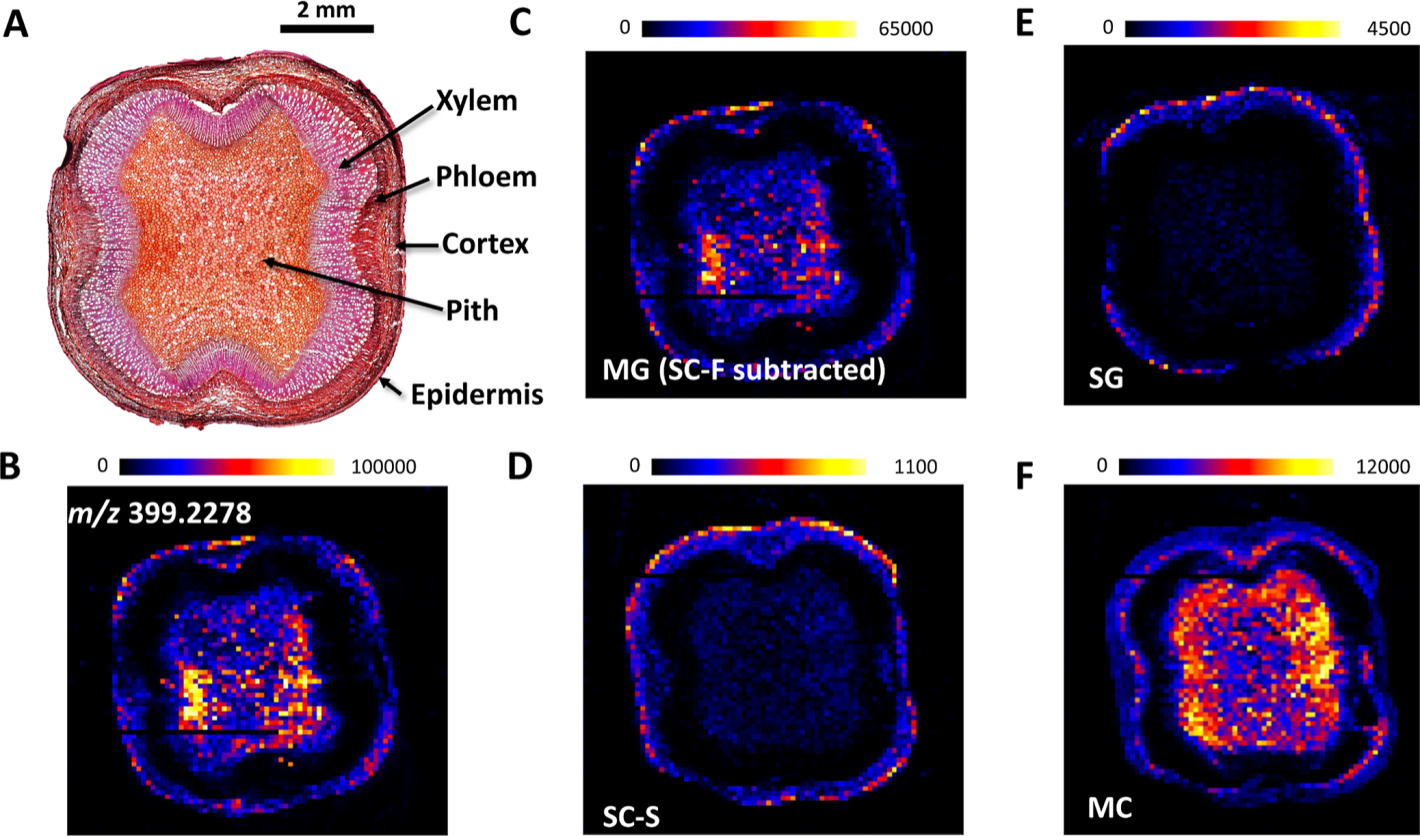
Stereoisomer-resolved mass spectrometry imaging (MSI) of kratom twig tissue using DESI-cIM-MS. (A) Light microscopy image of a kratom twig cross-section stained with toluidine blue and safranin O, highlighting key anatomical features including the cortex, vascular bundles, and central pith. (B) Ion image of *m*/*z* 399.2278 acquired without ion mobility separation, showing the combined spatial signal of all four mitragynine-type alkaloid diastereomers. (C–F) Ion maps of MG (after SC-F subtraction), SC-S, SG, and MC.

High-resolution DESI-cIM-MS imaging revealed distinct spatial compartmentalization of mitragynine-type stereoisomers within the *M. speciosa* twig. MG was widely distributed across the cortex and pith but was absent from the xylem, suggesting a general role throughout the tissue. SG and SC were concentrated mainly in the bark, especially the epidermis, implying specialized functions in outer protective layers. MC was primarily localized in the pith with some presence in the bark, indicating unique biosynthetic or storage roles. These patterns highlighted the plant’s intricate regulation of alkaloid distribution, likely reflecting differing biosynthesis, transport, and physiological functions. This spatial insight provided a foundation for future studies on alkaloid metabolism and their ecological and medicinal roles in kratom.

To extend our study beyond these stereoisomers, we investigated two pharmacologically important kratom alkaloids: paynantheine and 7-OH-mitragynine. Paynantheine is a partial μ-opioid receptor agonist with muscle relaxant properties,^23^ while 7-OH-mitragynine is a highly potent μ-opioid agonist with significantly greater efficacy than MG.^24^ In both cases, cIM-MS revealed isobaric interferences, molecules with nearly identical *m*/*z* values but different arrival times and spatial distributions (Figure 6). These distinctions, invisible to conventional MSI approaches, were clearly resolved using a 16-pass analysis, revealing that paynantheine and 7-OH-mitragynine are distributed throughout the twig except in the xylem.

**Figure 6:**
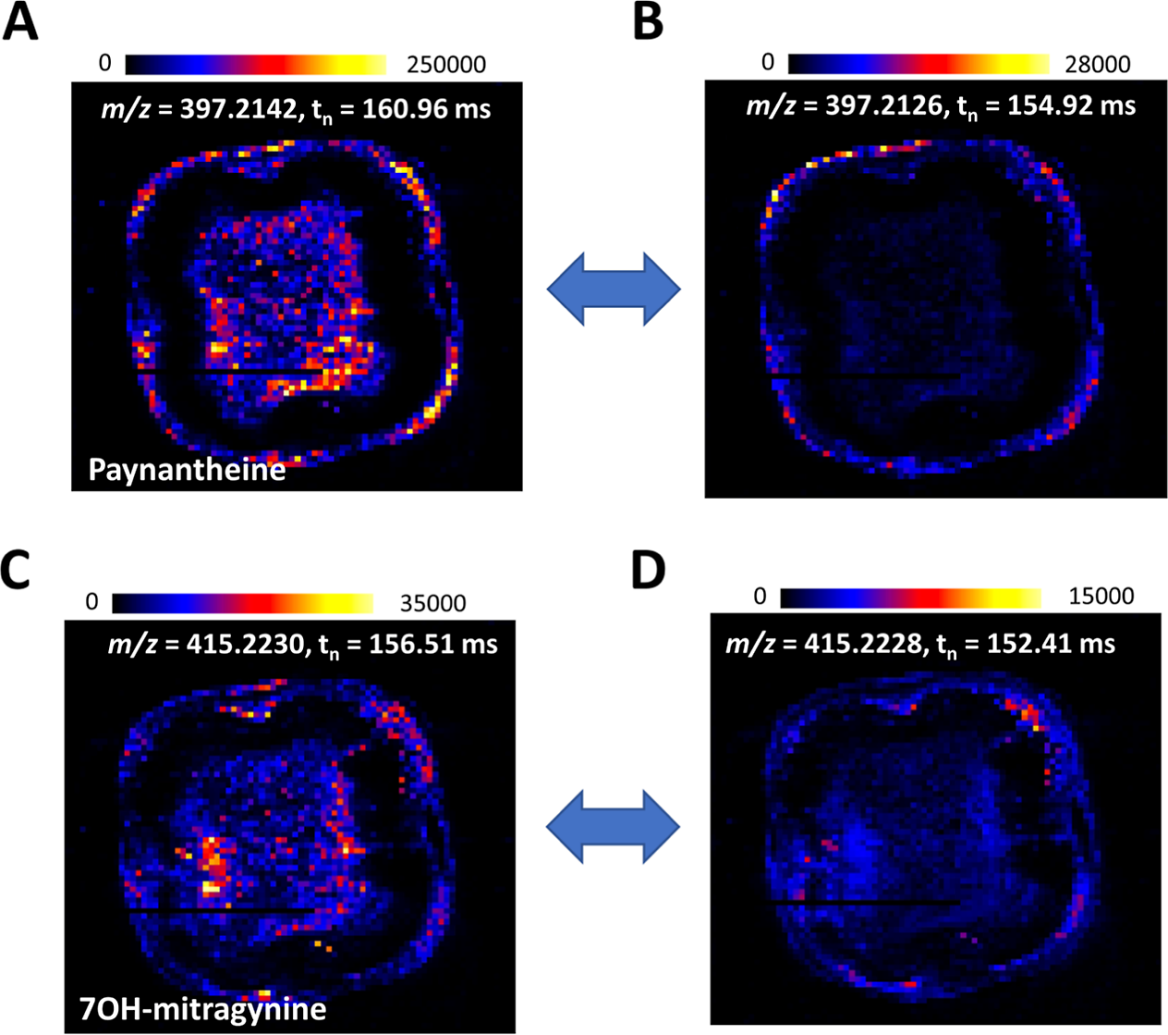
Distinct spatial distributions of important kratom alkaloids resolved by DESI-cIM-MS with a separation time of 141 ms. (A,B) Ion images of paynantheine (*m*/*z* 397.2142, *t*_*n*_ = 160.96 ms) and its isobaric counterpart (*m*/*z* 397.2126, *t*_*n*_ = 154.92 ms). (C–D) Ion images of 7-OH-mitragynine (*m*/*z* 415.2230, *t*_*n*_ = 156.51 ms) and a codetected isobar (*m*/*z* 415.2228, *t*_*n*_ = 152.41 ms).

Our findings demonstrate that integrating ultrahigh-resolution cyclic ion mobility into the DESI platform enables structural resolution and spatial separation of closely related small molecules directly in complex biological tissues. Unlike conventional DESI-MS, which merges isomeric species into a single ion image, our approach distinguishes subtle structural variants—such as conformers and diastereomers—that would otherwise remain unresolved. This level of specificity provides a more accurate view of spatial chemical organization and reveals biological heterogeneity that traditional MSI techniques may obscure. The ability to confidently annotate and localize stereoisomers and isobars in situ opens new avenues for investigating spatial metabolism, biosynthetic compartmentalization, and chemical ecology in plant systems and beyond.

## Conclusion

This study presents the first DESI-cIM-MS method enabling the in situ separation and spatial imaging of stereoisomeric small molecules directly in plant tissue. Using alkaloids from *M. speciosa* as a model system, we demonstrated the power of multipass cyclic ion mobility and targeted ion slicing to resolve closely related molecular species that would otherwise be indistinguishable in conventional MSI. The reproducible detection of stereoisomers and the dual-conformer profile of SC highlights the platform’s sensitivity and structural specificity. Although MG and SC-F remained unresolved under current conditions, our pixel-wise subtraction strategy mitigated this limitation. By extending MSI into the domain of stereochemical resolution, this method establishes a generalizable and label-free approach for spatial metabolomics, natural product discovery, and pharmacological analysis in complex biological matrices. As ion mobility resolution continues to improve, so too will our ability to fully dissect the structural complexity of small molecules in situ, opening new avenues for mechanistic insight into biosynthetic localization, tissue organization, and bioactivity.

## Supplementary Material



## Data Availability

This article contains Supporting Information in supplementary table. The data will be made available on request.

## Notes

Supplementary Figure S1 showing MS/MS spectra of the two gas-phase conformers of speciociliatine and supplementary tables reporting arrival times for the four stereoisomers and quantum chemical analysis of their gas-phase conformations (PDF)
