# Supplementary material for: Spatial Mapping of Stereoisomeric and Isobaric Alkaloids in Mitragyna speciosa Tissues by High-Resolution DESI-cIM-MS
Source: Anal Chem. 2025 Nov 14;97(47):26088–96. doi: 10.1021/acs.analchem.5c04730 (PMC12676509; doi:10.1021/acs.analchem.5c04730)
Supplement: Supplementary file 1 [file ac5c04730_si_001.pdf]

Supplementary Information

for

Spatial Mapping of Stereoisomeric and Isobaric Alkaloids in *Mitragyna speciosa* Tissues by High-Resolution DESI-cIM-MS

Pattipong Wisanpitayakorn[1,2,3], Adchata Konsue[1,2,3], Thanutchaporn Sartyoungkul[1,2,3], Ammarin In-on[1,2,3], Yongyut Sirivatanauksorn[2,3,], David R. Gang[4], Prasat Kittakoop[5,6], Sakda Khoomrung[1,2,3,7,8,*]

[1]Siriraj Center of Research Excellence in Metabolomics and Systems Biology (SiCORE-MSB), Faculty of Medicine Siriraj Hospital, Mahidol University, Bangkok 10700, Thailand

[2]Siriraj Metabolomics and Phenomics Center, Faculty of Medicine Siriraj Hospital, Mahidol University, Bangkok 10700, Thailand

[3]Thailand Metabolomics Association, Bangkok 10700, Thailand

[4]Institute of Biological Chemistry, Washington State, University, Pullman, WA, 99164, USA.

[5]Chulabhorn Graduate Institute, Program in Chemical Sciences, Chulabhorn Royal Academy, Laksi, Bangkok 10210, Thailand

[6]Chulabhorn Research Institute, Laksi, Bangkok 10210, Thailand

[7]Department of Biochemistry, Faculty of Medicine Siriraj Hospital, Mahidol University, Bangkok 10700, Thailand

[8]Center of Excellence for Innovation in Chemistry (PERCH-CIC), Faculty of Science Mahidol University, Bangkok 10400, Thailand

Table of Contents

- **Supplementary Figure S1.** MS/MS spectra of the two gas-phase conformers of speciociliatine (SC) acquired at a collision energy of 30 V: (A) fast conformer (SC-F) and (B) slow conformer (SC-S). – page S3
- **Supplementary Table S1.** Arrival times (milliseconds) across the first 20 passes and periodic drift time (tp, milliseconds) for the four mitragynine-type stereoisomers – page S4
- **Supplementary Table S2.** Computational investigation of QM conformation in the gas phase – page S4

Supplementary Figure S1. MS/MS spectra of the two gas-phase conformers of speciociliatine (SC) acquired at a collision energy of 30 V: (A) fast conformer (SC-F) and (B) slow conformer (SC-S).

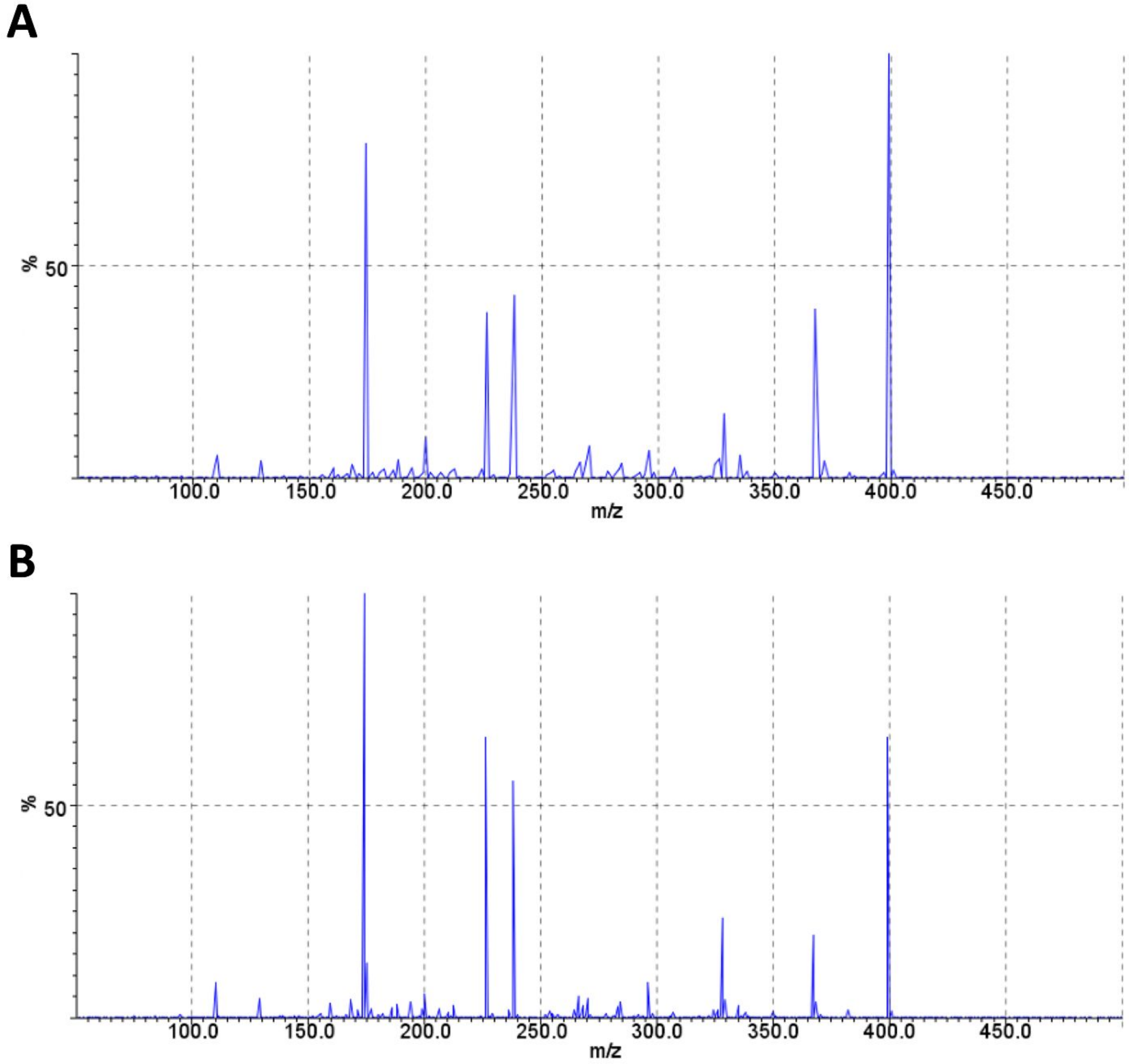

Supplementary Table S1. Arrival times (milliseconds) across the first 20 passes and periodic drift time (t_p, milliseconds) four mitragynine-type stereoisomers. Values represent mean arrival times (n = 3). The observed standard deviations were ≤ 0.01 ms for all compounds across all passes.

Pass Number	MC	MG	SC-F	SC-S	SG
**0**	11.59	11.57	11.61	11.61	11.65
**1**	20.59	20.37	20.71	20.71	20.58
**2**	29.48	29.09	28.16	29.77	29.40
**3**	38.39	37.76	37.81	38.73	38.26
**4**	47.38	46.55	46.64	47.89	47.16
**5**	56.33	55.26	55.40	56.86	56.02
**6**	65.40	64.06	64.22	65.96	64.97
**7**	74.37	72.84	73.10	75.08	73.93
**8**	83.33	81.61	81.90	84.26	82.80
**9**	92.34	90.43	90.76	93.38	91.76
**10**	101.39	99.27	99.61	102.52	100.62
**11**	110.35	108.13	108.39	111.60	109.55
**12**	119.41	116.83	117.19	120.69	118.53
**13**	128.42	125.66	126.07	129.80	127.40
**14**	137.41	134.49	134.84	138.87	136.35
**15**	146.42	143.22	143.71	148.08	145.23
**16**	155.39	152.01	152.51	157.10	154.19
**17**	164.49	160.74	161.40	166.26	163.14
**18**	173.50	169.68	170.25	175.50	172.08
**19**	182.49	178.43	178.94	184.42	181.03
**20**	191.51	187.15	187.80	193.56	189.95
**t_p**	**9.00**	**8.79**	**8.82**	**9.10**	**8.92**

Supplementary Table S2: Computational investigation of QM conformation in the gas phase.

ID	H-adduct on nitrogen	Energy (a.u)	Barrier (kcal/mol)
Speciociliatine	Downward	-1305.372369	0
	Upward	-1305.367894	2.80
Speciogynine	Downward	-1305.381261	-
	Upward	N/A	-
Mitraciliatine	Downward	-1305.389645	0
	Upward	-1305.373516	10.1
Mitragynine	Downward	-1305.377542	-
	Upward	N/A	-
